# Supplementary material for: Fourth-order kinematic analysis: Advanced decomposition methods for particle motion in modified orthogonal frame
Source: PLoS One. 2025 Dec 31;20(12):e0337900. doi: 10.1371/journal.pone.0337900 (PMC12755816; doi:10.1371/journal.pone.0337900)
Supplement: Attachment — Submitted filename: 13.09.2025.pdf [file pone.0337900.s001.pdf]

Referee Report

Title: Fourth-Order Kinematic Analysis: Advanced Decomposition Methods for Particle Motion in Modified Orthonormal Frame

Authors: Fatimah Alghamdi and Ayman Elsharkawy

The article under review explores decomposition methods for the fourth-order derivative of the position (snap vector) of a particle moving in three-dimensional Euclidean geometry along spatial curves. In-depth analytical expressions that break down the snap vector into parts that match modified orthonormal basis vectors are investigated. Additionally, an alternative formulation is introduced that divides the snap vector into radial and tangential components in the osculating and rectifying planes. Computational examples of the suggested decomposition strategies validate the created theoretical framework.

The results contribute to the broader field of differential geometry and kinematics. Additionally, the theme of the paper is relevant, the results appear interesting, mathematically sound, and significant from a scientific perspective. However, the main issue is an explanation of the motivation for the selection of a modified orthonormal frame, and minor issues are some simple grammatical errors

I suggest some modifications to correct or improve the text.

<u>Primary Concerns:</u>

1. In the last paragraphs of the Introduction, the handiness of modified frame applications was explained. However, it is not clear how this frame solves the problem that occurs when curves exhibit vanishing curvature. This must be clarified in the Preliminaries section, exactly when the formal definition of the modified orthogonal frame is given. For instance, **after Equations (2.5) (2.6), an expression is needed to ensure that curvature is not zero**; otherwise, undefined cases occur for the derivative formula. But we said the modified orthogonal frame solves the problem that occurs when curves exhibit vanishing curvature. I propose to check and refer to the paper doi.org/10.3390/axioms13110800. This issue was explained in detail in that paper.

2. In the given Example, a modified frame is not needed because the Frenet frame works for such a helix. We must understand how the new frame solves the deficiency in the example.

Minor Concerns:

1. On page 2, line 13: "**Th**" should be corrected as "The". In fact, the inner and cross products are well-known in Euclidean spaces, a more suitable entry may be selected to start.

2. The citations are absent on page 2. All information that is not your own must be cited.

3. On page 2, line 6: "The *modified orthogonal frame* along γ (u) defined as" should be corrected as "The *modified orthogonal frame* along γ (u) **is** defined as"

4. On page 2, line 10: "For the relationship the modified frame and Frenet frame, The *relation matrix* along γ (u) given by" should be corrected as "For the relationship **between** the modified frame and Frenet frame, **t**he *relation matrix* along γ (u) **is** given by"

5. In Theorem 1 and in proof: "synchronize" should be "synchronize**s"**

6. Theorem 2 and Corollary 1: it is unusual to give an equation number to an assumption.

7. On page 8, Proof: What does it mean "Assume that P in Figure 1 moves along γ (u)". Do you mean "Assume that P moves along γ (u) as can be seen in Figure 1 "

8. On page 8, equation (3.9): Put a full stop at the end of the equation.

9. It is recommended that Figures 2 and 3 be slightly enlarged to enhance readability.

In conclusion, the reviewed paper is **acceptable** for publication at **PLOS One** after thoroughly addressing the abovementioned **minor revision**s.
