# Supplementary material for: Fourth-order kinematic analysis: Advanced decomposition methods for particle motion in modified orthogonal frame
Source: PLoS One. 2025 Dec 31;20(12):e0337900. doi: 10.1371/journal.pone.0337900 (PMC12755816; doi:10.1371/journal.pone.0337900)
Supplement: Attachment — Submitted filename: Response to reviewers.pdf [file pone.0337900.s002.pdf]

Response to Reviewers Comments

We thank the Reviewers for their thoughtful feedback and valuable suggestions for improving our manuscript. We have carefully considered all comments and have made substantial revisions to address them. Below, we provide a point-by-point response to each comment.

Reviewer 1:

Comment 1: *"The abstract jumps directly into the aim and content of the paper. I recommend adding a sentence at the beginning to introduce the general topic and its potential applications."*

Response: We have added an introductory sentence at the beginning of the abstract to provide better context:

"The analysis of higher-order derivatives of position, particularly the snap (fourth derivative), provides critical insights into complex motion dynamics in fields such as robotics, aerospace, and biomechanics."

Comment 2: *"I suggest briefly explaining the concept of the Frenet frame to support readers who may be less familiar with it."*

Response: We have added an explanatory paragraph in the Introduction (Section 1) that briefly describes the Frenet frame:

"The Frenet frame consists of three orthonormal vectors: the tangent vector (indicating instantaneous direction of motion), the normal vector (pointing toward the center of curvature), and the binormal vector (orthogonal to both)."

Comment 3: *"The authors should more clearly emphasize the novelty of their contribution in light of existing gaps in the literature."*

Response: We have added a dedicated paragraph in the Introduction highlighting the specific gaps our research addresses:

"Our research addresses several gaps in the existing literature by: (1) providing the first comprehensive decomposition of the fourth-order snap vector using modified orthonormal frames, (2) introducing an innovative tangential-radial decomposition approach that offers new geometric insights, and (3) developing formulations that remain valid even when traditional Frenet frames become undefined due to vanishing curvature."

Comment 4: *"Sections 2 and 3 would benefit from introductory sentences, similar to the one provided at the beginning of Section 4."*

Response: We have added introductory paragraphs at the beginning of both Sections 2 and 3:

- **Section 2:** "This section establishes the fundamental mathematical framework and notation necessary for our subsequent analysis. We review key concepts from differential geometry, including curve parameterization, Frenet frames, and modified orthonormal frames, which form the foundation for our decomposition methods."
- **Section 3:** "In this section, we present our principal theoretical contributions: two novel decomposition theorems for the snap vector. The first theorem provides a decomposition using the modified orthonormal frame, while the second theorem offers an alternative formulation using tangential and radial components."

Comment 5: *"I recommend adding a final comment that discusses the relevance and implications of the application presented in Section 4."*

Response: We have added a concluding paragraph in Section 4 that discusses the practical implications:

"This application highlights how our approach can be implemented computationally and provides insights into motion analysis for engineering applications such as robotic path planning, vehicle dynamics, and animation systems where higher-order smoothness is crucial."

Comment 6: *"The conclusions should be expanded to better highlight the strengths of the proposed method, particularly in comparison with existing approaches."*

Response: We have completely rewritten the Conclusion section (Section 5) to:

- Explicitly compare our method with existing approaches
- Highlight the mathematical stability advantages over traditional Frenet frames
- Emphasize the dual decomposition approach as a key strength
- Discuss potential applications and future research directions

<u>Reviewer 2:</u>

Comment 1: *"Technical Error in Citation: In the Introduction, there is a technical error in the citation of references – [16, 17, 18, 19, 16]. The reference [16] is repeated and should be corrected."*

Response: We have corrected this citation error. The duplicate reference [16] has been removed from the citation list in the Introduction.

Comment 2: *"Missing Formula: Formula (3.4) is missing in the manuscript and should be included or corrected."*

Response: We have identified and corrected this oversight.

Comment 3: *"Overstated Contribution: The claim in the Introduction about the study's contribution to higher-order kinematic analysis appears to be overstated... A more accurate claim would be: 'This paper contributes to the presentation and further development of analytical techniques and alternative decomposition approaches in higher-order kinematic analysis.'"*

Response: We have revised the statement as suggested to provide a more accurate description of our contribution:

"This paper contributes to the presentation and further development of analytical techniques and alternative decomposition approaches in higher-order kinematic analysis."

Comment 4: *"Lack of Proper Acknowledgment of Prior Work: Sections 2 (Preliminaries) and 3 (Main Results) closely follow the methodology, analytical techniques, and decomposition approaches outlined in Hamouda [15], with only minor differences in parameter designations... These sections should begin with a clear statement that the work builds upon the methods developed in Hamouda [15]."*

Response: We have added explicit acknowledgments at the beginning of both Sections 2 and 3 that:

- Clearly state our work builds upon the methodology developed by Hamouda et al. [15]
- Outline the key differences in our approach (use of modified orthonormal frames vs. quasi-frames)
- Highlight the advantages of our modified frame approach, particularly regarding mathematical stability

Comment 5: *"Unacknowledged Use of Prior Example: In Section 4 – Application, the example of a particle moving along a helical curve (Fig.3) is used to illustrate the results. However, this example was already presented by Guner [13], and this should be clearly acknowledged."*

Response: We have added a clear acknowledgment at the beginning of Section 4 that:

- Explicitly references Guner [13] for the helical curve example
- Explains the differences in our application (snap vector vs. jerk vector analysis, modified frames vs. Frenet frames)
- Highlights how our results provide complementary insights

Reviewer 3:

Major Comment 1: *"In the last paragraphs of the Introduction, the handiness of modified frame applications was explained. However, it is not clear how this frame solves the problem that occurs when curves exhibit vanishing curvature. This must be clarified in the Preliminaries section, exactly when the formal definition of the modified orthogonal frame is given."*

Response: We have added a detailed clarification in the Preliminaries section (after Equation 2.6) that explicitly explains how the modified frame addresses the vanishing curvature problem. The added text states:

"The modified orthogonal frame addresses a critical limitation of the Frenet frame: it remains well-defined even when curves exhibit vanishing curvature. While the Frenet frame becomes undefined when $\kappa=0$ (as $N=T'/\|T'\|$ becomes indeterminate), the modified frame vectors $N_m=T'$ and $B_m=T\times T'$ remain mathematically valid regardless of curvature values."

Major Comment 2: *"In the given Example, a modified frame is not needed because the Frenet frame works for such a helix. We must understand how the new frame solves the deficiency in the example."*

Response: We have added an explanation in the Application section clarifying our rationale:

"While the Frenet frame is well-defined for this helix (since $\kappa\neq0$), we employ the modified orthogonal frame to demonstrate the consistency of our decomposition methodology and to provide a foundation for future applications where Frenet frames may become problematic. This example serves as a validation case where both frames are applicable, allowing readers to compare results and build intuition before applying these methods to more complex curves with potential curvature singularities."

Minor Concerns - All Addressed:

1. **"Th" correction:** Changed "Th inner" to "the inner product and vector cross product" in the Preliminaries section.
2. **Missing citations:** Added appropriate citations for the definitions of regular curves and inner/cross products.
3. **Grammar correction:** Changed "defined as" to "is defined as" for the modified orthogonal frame definition.
4. **Grammar correction:** Changed "For the relationship the modified frame" to "For the relationship between the modified frame" and corrected capitalization.
5. **Verb agreement:** Changed "synchronize" to "synchronizes" in Theorem 1 and its proof.

6. **Equation numbering:** Removed the equation number from the assumption in Theorem 2 and Corollary 1.
7. **Figure reference clarity:** Changed "Assume that P in Figure 1" to "Assume that P moves along $\gamma(u)$ as shown in Figure 1".
8. **Punctuation:** Added a period at the end of Equation (3.9).
9. **Figure sizing:** Increased the size of Figures 2 and 3 from 0.4/0.2 textwidth to 0.5/0.3 textwidth respectively to enhance readability.

We believe these revisions have significantly improved the clarity and rigor of our manuscript. The added explanations provide better context for the modified frame's advantages, and the grammatical corrections enhance the overall readability. We thank the reviewers for their detailed feedback that has helped strengthen our paper.
