# Supplementary material for: Fourth-order kinematic analysis: Advanced decomposition methods for particle motion in modified orthogonal frame
Source: PLoS One. 2025 Dec 31;20(12):e0337900. doi: 10.1371/journal.pone.0337900 (PMC12755816; doi:10.1371/journal.pone.0337900)
Supplement: Attachment — Submitted filename: reply round 2.pdf [file pone.0337900.s003.pdf]

1 Response to Reviewer Comments

We thank the reviewer for their insightful comments and suggestions. We have carefully addressed each point as follows:

Comment 1: Clarification on Vanishing Curvature

"The authors responded that 'We have added a detailed clarification in the Preliminaries section (after Equation 2.6) that explicitly explains how the modified frame addresses the vanishing curvature problem.' But the problem is that if $\kappa = 0$, how could Eq. (2.5) or (2.6) be possible? The reviewer still proposes that authors focus on the previous suggestion."

Response: We appreciate this important clarification. We have now added a detailed explanation in the Preliminaries section (after Equation 2.6) that addresses this concern directly. The key points are:

1. The modified orthogonal frame $\{\mathbf{T}, \mathbf{N_m}, \mathbf{B_m}\}$ is defined *directly* from the curve derivatives: $\mathbf{N_m} = \mathbf{T}'$ and $\mathbf{B_m} = \mathbf{T} \times \mathbf{T}'$, independent of curvature.
2. Equations (2.5) and (2.6) are derived under the assumption $\kappa \neq 0$ specifically to establish the relationship between modified and Frenet frames. However, the modified frame itself remains mathematically valid when $\kappa = 0$.
3. When $\kappa = 0$, the derivative formulas would need to be derived separately without relying on the Frenet relations, but the frame vectors themselves remain well-defined.

This clarification emphasizes that the modified frame's primary advantage is its definitional robustness, while the specific equations (2.5)-(2.6) represent special cases for non-vanishing curvature.

Comment 2: Example Selection

"The reviewer still believes that the example must be chosen for a curve where the Frenet frame does not work. Then, referring to the Modified Orthogonal Frame may be meaningful. Otherwise, the readers cannot sense the benefit of MOF in the authors' theoretical results."

Response: We completely agree with this suggestion. We have now added another example of a curve where the Frenet frame does not work, but the Modified Orthogonal frame is defined.

Comment 3: Terminology Consistency

"In title and elsewhere, 'modified orthonormal frame' or 'modified orthogonal frame'?"

Response: Thank you for catching this inconsistency. We have standardized the terminology throughout the paper to use **"modified orthogonal frame"** because:

- The vectors $\mathbf{T}$, $\mathbf{N_m}$, and $\mathbf{B_m}$ are orthogonal but not necessarily orthonormal, since $\mathbf{N_m} = \mathbf{T}'$ is not normalized.
- We have updated the title and all instances in the text to maintain consistency.

These revisions significantly strengthen our paper by clearly demonstrating the modified orthogonal frame's advantages and providing concrete examples where it outperforms traditional Frenet analysis.
